# Depression and Anxiety During Pregnancy in Mexican Women: Prevalence and Associated Factors in the OBESO Cohort Study

**DOI:** 10.3390/jcm14238364

**Published:** 2025-11-25

**Authors:** Blanca Vianey Suárez-Rico, Isabel González-Ludlow, Jonatan Alejandro Mendoza-Ortega, Guadalupe Estrada-Gutierrez, Pilar de Abiega-Franyutti, Salvador Espino y Sosa, Maria del Carmen Hernández-Chávez, Erika Osorio-Valencia, Gabriela Gil-Martínez, Araceli Montoya-Estrada, José Romo-Yañez, Ignacio Camacho-Arroyo, Otilia Perichart-Perera, Enrique Reyes-Muñoz

**Affiliations:** 1 Department of Immunobiochemistry, National Institute of Perinatology, Mexico City 11000, Mexico; blancasuarezrico@gmail.com (B.V.S.-R.); jonatan.mdz93@gmail.com (J.A.M.-O.); guadalupe.estrada@inper.gob.mx (G.E.-G.); pilardeabiega@gmail.com (P.d.A.-F.); 2 División de Doctorado en Psicología, Instituto Suizo Universidad, Puebla 72160, Mexico; 3 Department of Nutrition and Bio Programming, Instituto Nacional de Perinatología Isidro Espinosa de los Reyes, Mexico City 11000, Mexico; isaglezludlow@icloud.com (I.G.-L.); otiliaperichart@inper.gob.mx (O.P.-P.); 4 Science and Health Faculty, Campus Norte, Universidad Anáhuac México, Huixquilucan 52786, Mexico; 5 Clinical Research Branch, Instituto Nacional de Perinatología Isidro Espinosa de los Reyes, Mexico City 11000, Mexico; salvadorespino@gmail.com; 6 Department of Developmental Neurobiology, Instituto Nacional de Perinatología Isidro Espinosa de los Reyes, Mexico City 11000, Mexico; karmenhdez@hotmail.com (M.d.C.H.-C.); erika.osorio@inper.gob.mx (E.O.-V.); geemege2001@yahoo.com.mx (G.G.-M.); 7 Coordination of Gynecological and Perinatal Endocrinology, Instituto Nacional de Perinatología Isidro Espinosa de los Reyes, Mexico City 11000, Mexico; ara_mones@hotmail.com (A.M.-E.); jryz@yahoo.com (J.R.-Y.); 8 Unidad de Investigación en Reproducción Humana, Instituto Nacional de Perinatología-Facultad de Química, Universidad Nacional Autónoma de México, Mexico City 02090, Mexico; ignacio.camacho@inper.gob.mx

**Keywords:** anxiety, depression, pregnancy, prenatal, perinatal

## Abstract

**Background:** Depression and anxiety during pregnancy are global public health issues, as both are linked to adverse outcomes for mothers and their newborns. This study aimed to determine the prevalence of depression and anxiety during pregnancy and identify the factors associated with these conditions in Mexican women. **Methods:** This prospective cohort study included 288 participants. The Edinburgh Postnatal Depression Scale and the State-Trait Anxiety Inventory were applied during the second trimester of pregnancy. The factors associated with depression and anxiety were analyzed. **Results:** The prevalence of depression and anxiety was 20.5% (95% confidence interval (CI) 16.1–25.7) and 22.2% (95% CI 17.8–27.3), respectively. The factors independently associated with an increased risk of depression, expressed as adjusted odds ratios with (95% CI), were anxiety, 10.8 (5.4–21.6), *p* = 0.0001, and educational level, secondary school and high school, 2.6 (1.10–6.14). In contrast, a bachelor’s degree was associated with a lower risk of depression, 0.38 (0.16–0.90), *p* = 0.03. The factors independently associated with a higher risk of anxiety were depression, 11.2 (5.6–22.1), and having children, 3.1 (1.6–6.1), *p* = 0.03. **Conclusions:** Our results show that one in five pregnant women exhibit clinical symptoms of depression or anxiety, and one in eight women have both conditions simultaneously. These findings highlight the importance of routine mental health screening and assessment during pregnancy. The factors linked to depression include anxiety and having completed secondary or high school education, while depression and having children were associated with anxiety.

## 1. Introduction

Depression and anxiety during pregnancy represent a global public health issue, as they cause both short-term and long-term adverse outcomes for the mother and the newborn [1,2]. Prenatal depression has been identified as a risk factor for various complications during childbirth, which can result in infant morbidity and mortality [1,2,3,4,5]. Although depression is the most common mental disorder during pregnancy, it is less studied than postpartum depression, despite its significant consequences for both woman and child [5,6].

Maternal mental health can impact the quality of the fetal environment in several ways. The first is an indirect pathway where stress or distressing mental states trigger physiological responses, such as endocrine, metabolic, or immune reactions, which are then transmitted across the placenta to the fetus [7]. The second pathway involves a woman’s mental state influencing her engagement in healthy behaviors during pregnancy. For example, depression and anxiety during pregnancy appear to be related to levels of physical activity [8], and mental health concerns often occur with substance use disorders [8,9]. A third pathway involves the connection between mental health and pregnancy-related medical complications such as hypertension, gestational diabetes mellitus, fetal growth restriction, and viral infection [7,8,9,10,11].

Anxiety during pregnancy and the postnatal period has been linked to several negative outcomes for both women and their children [12,13]. Women experiencing perinatal anxiety and depression often use less effective coping strategies, which can lead to fear of childbirth, pregnancy-related phobias (tokophobia), and in extreme cases, even suicide [12,14,15,16]. Additionally, anxiety and depression could affect the baby’s health by increasing the risk of preterm birth and contributing to poor cognitive, emotional, and behavioral development during childhood and into adulthood [12,14,15,16,17,18,19,20].

In a meta-analysis of 23 observational studies involving a total of 25,663 women, untreated depression was associated with significantly higher risks of preterm birth and low birth weight, with a trend toward increased risks for more severe depression [5]. In another meta-analysis that included 195,751 unique mother-child dyads, maternal perinatal depression and anxiety were linked to poorer social-emotional, cognitive, language, motor, and adaptive behavior development in offspring during the postnatal period in developing countries [15].

A meta-analysis that examined the prevalence of depression among women living in low- and middle-income countries during pregnancy and up to one year postpartum found that depression affects one in four women during the perinatal period [14]. Likewise, it was concluded that accurate prevalence estimates are essential for guiding public policy and informing future research to improve outcomes for women, infants, and families [14].

Roddy Mitchell et al. also documented the prevalence of six anxiety disorders among women in low- and middle-income countries. Generalized anxiety disorder was the most common, affecting about one in five women during the perinatal period [12]. This meta-analysis highlights that anxiety disorders are nearly as common as antenatal depression, yet they receive significantly less attention [12]. Notably, this report included only one study that examined antenatal anxiety in Mexican women [21].

In a study involving Chinese women, being a housewife or unemployed, primiparity, the presence of stress or depression, and low levels of social support were linked to symptoms of anxiety. Conversely, engaging in physical exercise was identified as a protective factor. Concerning prenatal depression, associated factors included anxiety, lack of spousal support, low or moderate family care, and limited social support [22]. Another review found that low educational attainment, limited income, unplanned pregnancy, a history of psychological disorders and depression, as well as the presence of anxiety, stress, or poor social support during pregnancy, were linked to prenatal depression [23].

There is an urgent need to understand and address issues related to perinatal mental health, especially anxiety and depression, which can greatly affect both the mother and the baby. This study aimed to evaluate the related factors and the prevalence of anxiety and depression during pregnancy in Mexican women.

## 2. Materials and Methods

### 2.1. Study Design

This study is part of OBESO (Epigenetic and Biochemical Origin of Overweight and Obesity), a prospective perinatal cohort conducted at the Instituto Nacional de Perinatología Isidro Espinosa de los Reyes (INPerIER) in Mexico City. The OBESO cohort examines how maternal nutrition, lifestyle, mental health, metabolic, and inflammatory profiles impact pregnancy outcomes, neurodevelopment, and body composition of offspring. This prospective cohort study was conducted from 2017 to 2021. The protocol followed the ethical principles of the Declaration of Helsinki for medical research involving human subjects. All participants provided written informed consent before enrollment. The protocol was approved by the Research and Ethics Committee of INPerIER in Mexico City (protocol number: 33300-11-402-01-575-17, and its continuation, register number 2024-1-14).

### 2.2. Participants and Procedures

Participants were invited to join the OBESO Cohort during their first-trimester ultrasound, between 11 and 13.6 weeks of gestation. The inclusion criteria included pregnant women over 18 years old who were receiving prenatal care at INPerIER and agreed to complete mental health questionnaires. Exclusion criteria comprised a history of anxiety or depression, current use of medication for these conditions, and comorbidities such as pregestational diabetes, chronic hypertension, renal, psychiatric, cardiac, autoimmune, neurological, and hematological disorders. Demographic and clinical information were collected from participants’ medical records.

To assess symptoms of depression and anxiety during the second trimester of pregnancy (weeks 20 to 24), the Edinburgh Postnatal Depression Scale (EPDS) [24] and the State-Trait Anxiety Inventory (STAI) [25] were utilized. The EPDS is a 10-item, self-report questionnaire with scores ranging from 0 to 30, and it has previously been validated for use in the Mexican population [26,27]. The reliability of the EPDS in this study was excellent (Cronbach’s alpha = 0.88). The STAI was employed to assess anxiety levels and is a standardized, validated self-report instrument used within the Mexican population [28]. The STAI includes two separate self-rating scales measuring different dimensions of anxiety. The State Anxiety Scale (S-STAI) consists of 20 statements, requiring respondents to indicate how they feel at the moment of completing the scale. Scores on the S-STAI range from 20 to 80. A cutoff score above 40 indicates clinically relevant anxiety symptoms during the perinatal period, as recommended [29]. In this study, the Cronbach’s alpha was 0.92 for the STAI scale.

### 2.3. Study Variables

Depression was defined as an EPDS score of 13 or higher [26], while anxiety was indicated by a score of 40 or above on the S-STAI [29]. The evaluated associated risk factors for anxiety and depression included maternal age (grouped into three categories: <25, 25–34, and >34 years), pregestational body mass index (BMI) (normal: 18.5–24.99 kg/m^2^ overweight: 25–29.99 kg/m^2^; obesity: >30 kg/m^2^), unemployment status, marital status (married, cohabiting, and single), educational level (secondary school, high school, and bachelor’s degree), physical activity (more than 150 min per week), number of pregnancies, unplanned pregnancy, history of live births, history of preterm birth, spontaneous abortion, perinatal death, and the presence of nausea and vomiting three or more days per week during the first trimester.

### 2.4. Sample Size

The sample size was calculated to detect a 20% prevalence of depression during pregnancy, with a 95% confidence interval and a 5% margin of error. Based on these parameters, the minimum required sample size was 246 women. We included 288 women who met the inclusion criteria throughout the study period. 

### 2.5. Statistical Analysis

The prevalence of depression and anxiety was estimated with 95% confidence intervals (CIs). Continuous variables are expressed as means and standard deviations, while categorical variables are presented as frequencies and proportions. Student’s *t*-test or the Mann–Whitney U test was used to compare continuous variables, depending on data distribution; chi-square or Fisher’s exact test was used to evaluate differences in proportions. A *p*-value < 0.05 was considered statistically significant.

Subsequently, a contingency table was used to calculate odds ratios (ORs) and 95% CIs for each variable linked to depression and anxiety. Lastly, a logistic regression analysis was performed to assess factors independently associated with the risk of depression and anxiety in the bivariate analysis, reporting adjusted odds ratios (aORs) with 95% CIs. Statistical analyses were carried out using SPSS software, version 24 (IBM Corp., Armonk, NY, USA).

### 2.6. Use of Generative Artificial Intelligence Tools

During the writing of this manuscript, the authors used Grammarly [https://www.grammarly.com, accessed on 4 October 2025], San Francisco, CA, USA, and ChatGPT (version GPT-5, OpenAI, San Francisco, CA, USA) for grammar and editing revisions. The authors have reviewed and edited the output and assume full responsibility for the content of this publication.

## 3. Results

**Figure 1:**
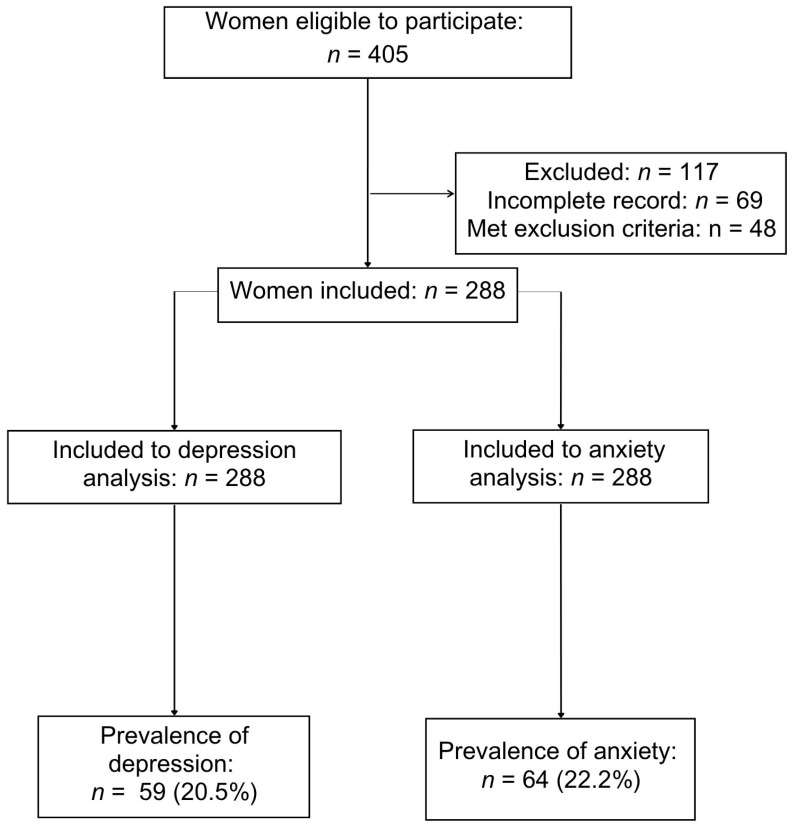
Flowchart of participants in the study.

Out of 405 eligible women, 288 participants were included in the study (Figure 1). The prevalence of depression was 20.5% (95% CI: 16.1–25.7), the prevalence of anxiety was 22.2% (95% CI: 17.8–27.3), and the prevalence of both anxiety and depression was 12.8% (95% CI: 9.7–17.2). The average score on the EPDS scale was 7.3 ± 6.1, and on the S-STAI scale was 34.4 ± 10.2.

### 3.1. Characteristics of Participants

Table 1 shows the characteristics of our study population. The mean maternal age was 30 years, the mean pre-pregnancy BMI was 27.6, and the mean gestational age at enrollment was 13 weeks. In terms of education, 21.1% of participants completed secondary school, 42.2% finished high school, and 36.7% earned a bachelor’s degree or higher.

Among the 288 participants, 39.6% were married, 38.9% were cohabiting, and 21.5% were single. Regarding pregestational BMI, 37.2% had a normal weight, 36.8% were overweight, and 26% were classified as obese.

### 3.2. Factors Associated with Depression

**Figure 2:**
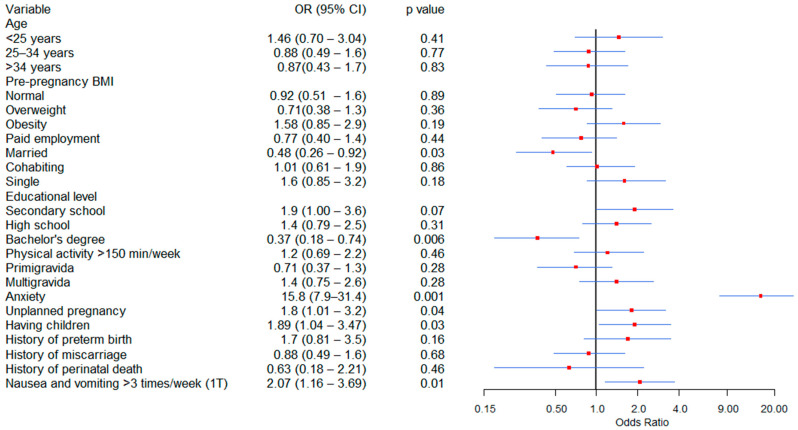
Factors Associated with Depression During Pregnancy: Bivariate Analysis. BMI = Body mass index, 1T = Fisrt trimester.

Figure 2 shows the factors associated with depression in the bivariate analysis; anxiety, unplanned pregnancy, having children, and experiencing nausea and vomiting more than three times weekly during the first trimester were significantly associated with a higher risk of depression. Conversely, being married and holding a bachelor’s degree were associated with protective factors against depression.

Table 2 shows the results of the logistic regression analysis. The factors that remained independently associated with depression after adjusting for marital status, secondary and high school education, having a bachelor’s degree, anxiety, unplanned pregnancy, having children, and experiencing vomiting more than three times weekly during the first trimester were: secondary and high school education levels; aOR 2.60 (95% CI 1.10–6.14), *p* = 0.03; anxiety; aOR 10.8 (95% CI 5.4–21.6), *p* = 0.0001; meanwhile, a bachelor’s degree was linked to a protective factor against depression; aOR 0.38 (95% CI 0.16–0.90), *p* = 0.03.

### 3.3. Factors Associated with Anxiety

**Figure 3:**
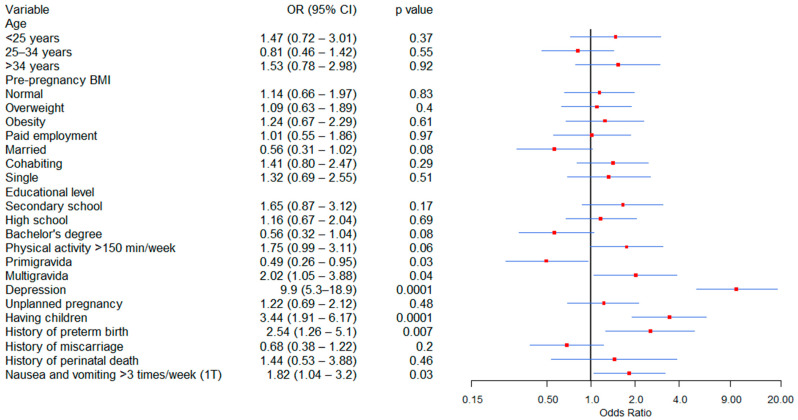
Factors Associated with Anxiety During Pregnancy: Bivariate Analysis. BMI = Body mass index, 1T = First trimester.

Figure 3 shows the factors associated with anxiety in the bivariate analysis; multigravida, depression, having living children, a history of preterm birth, and experiencing nausea and vomiting more than three times per week during the first trimester were significantly linked to an increased risk of anxiety. Conversely, being a primigravida was significantly associated with a protective factor against anxiety.

Table 3 shows the results of a logistic regression analysis examining factors associated with anxiety. The factors that remained independently associated with anxiety after adjusting for key variables identified in the bivariate analysis were depression, with an aOR of 11.2 (95% CI 5.6–22.1), *p* = 0.0001, and having living children, with an aOR of 3.1 (95% CI 1.6–6.1), *p* = 0.03.

The remaining evaluated factors—age, pregestational body mass index, employment status, educational level, physical activity >150 min per week, number of pregnancies, unplanned pregnancy, history of preterm birth, miscarriage, perinatal death, and nausea and vomiting >3 times/week—were not associated with either anxiety or depression in this study population.

## 4. Discussion

### 4.1. Principal Findings

In this group of pregnant women in Mexico during the second trimester, we found that the prevalence of depression was 20.5% and anxiety was 22.2%; co-occurring depression and anxiety were present in 12.8%. In a logistic regression analysis, the factors independently associated with an increased risk of depression were anxiety (aOR 10.8; 95% CI 5.4–21.6), and having a secondary or high school education (aOR 2.60; 95% CI 1.10–6.14). Meanwhile, a bachelor’s degree was linked to a protective factor against depression; aOR 0.38 (95% CI 0.16–0.90), *p* = 0.03. On the other hand, independent factors that increase the risk of anxiety included depression (aOR 11.2; 95% CI 5.6–22.1) and having children (aOR 3.1; 95% CI 1.6–6.1).

### 4.2. Comparison with Existing Literature

These findings on depression prevalence are consistent with those reported in the meta-analysis by Rody Mitchell et al. [14], which documented a prevalence of perinatal depression of 21.7% (95% CI 20.1–23.2) across 90 studies conducted in Latin American and Caribbean countries. This result is similar to our findings. However, the depression rate in our study was higher than that reported by Shakeel et al. in a prospective cohort of 749 pregnant women attending early antenatal care in Oslo. They found depression prevalences of 8.6% (95% CI: 5.45–11.75%) in Western Europeans, 19.5% (95% CI: 12.19–26.81%) in Middle Easterners, and 17.5% (95% CI: 12.08–22.92%) in South Asians [30].

The prevalence of anxiety observed in this study (22.2%) was similar to that in the study of Nielsen-Scott et al., who estimated a pooled prevalence of self-reported anxiety symptoms of 24.4% (95% CI: 16.2–33.7%) in low- and middle-income countries [13], likewise, the prevalence of anxiety during pregnancy in this study is similar to 20.8% (95% CI 16–26) reported in 30 studies performed in Latin America and the Caribbean countries, but is significantly higher than that reported in Poland of 7.7%, and lower than that found in Italy of 36.5%, using the same cut-off of the S-STAI questionnaire [6].

A previous study among women in Mexico City using the 10-item anxiety subscale from the Symptoms Checklist-90 reported a 21% prevalence of anxiety during the third trimester of pregnancy [21], which aligns with our findings using the STAI questionnaire.

Regarding risk factors associated with depression and anxiety, this study found that anxiety and having a secondary or high school education were associated with depression symptoms after adjustment for significant variables identified in the bivariate analysis. Like our findings, the study by Tang et al., conducted in a Chinese population, reported that a group-oriented personality type, the presence of anxiety, and the lack of partner support were factors associated with depression [22]. Additionally, low or moderate family care and low social support were also connected to depression. Differences in the scales used and variations in cultural and contextual factors may partly explain these discrepancies [22].

A systematic review examined the factors linked to depression during the perinatal period. The most reported risk factor associated with high depressive symptom trajectories was a history of depression before pregnancy [23]. However, we excluded women with a prior history of depression from our study cohort, which limited our ability to assess this risk. Other associated factors included being young, belonging to an ethnic minority, low maternal education, low income, being single or facing relationship issues, unplanned pregnancy, and high stress levels [23]. Similarly, Yang et al. found that low educational attainment, low-income family situations, a history of mental illness, domestic violence, tobacco or alcohol use during the perinatal period, and multiparity are risk factors for perinatal depression [31]. In our study, anxiety was the most significant factor associated with depression, but several other studies that examined risk factors related to depression during pregnancy did not assess anxiety. This likely explains why anxiety has not been previously reported as a factor associated with depression in these systematic reviews. Conversely, in our study, having a bachelor’s degree was an independent protective factor against depression, aligning with a systematic review that identified positive social support and higher education levels as protective factors [32].

A meta-analysis conducted by Yin et al. found that a history of depression, low social support, being single, separated, or divorced, unplanned pregnancy, unemployment, exposure to violence, and tobacco use before or during pregnancy were significantly associated with prenatal depression [33]. Among these factors, being single and an unplanned pregnancy were notably associated with depression in our study [33]. This symptomatology may not only reflect physiological changes but also worsen the perceived loss of control, reduced functioning, and increased stress, as previously reported by Mitchel et al. [14]. It is essential to note that the meta-analysis above included global data from countries with varying income levels, encompassing low-, middle-, and high-income nations [33]. In contrast, the current study was conducted solely within a Mexican population.

On the other hand, the study by Tang et al. identified risk factors for prenatal anxiety, such as being a housewife (without paid employment), primiparity, stress, depression, and low levels of social support [22]. At the same time, physical exercise was found to be a protective factor. In contrast, in the present study, the risk factors associated with anxiety were depression and having children. Although unpaid work, physical activity, and primiparity were evaluated, none of these factors were significantly associated with anxiety. Additionally, this study did not assess social support levels.

### 4.3. Strengths and Limitations

The strengths of this study include its sample size with sufficient power to estimate the prevalence of depression and anxiety during pregnancy using validated questionnaires for Mexican women, and its cohort design, which reduces the possibility of bias.

This study has some limitations. Relying on self-report instruments may introduce response bias. Additionally, excluding women with a history of diagnosed depression or anxiety might have led to an underestimation of the true prevalence. Since the sample was recruited from a tertiary care center, the findings may not be generalizable to other settings in Mexico.

In this study, women with a history of anxiety and depression were excluded, which may lead to an underestimation of the true prevalence of depression and anxiety during pregnancy. However, it highlights the importance of screening women who are unaware they have anxiety or depression to identify and address these issues during pregnancy.

### 4.4. Clinical Implications

These findings highlight the importance of including mental health screening as part of routine prenatal care. Using validated tools, such as the EPDS and STAI, facilitates early detection and enables medical referral to appropriate psychological care. Additionally, public health strategies should involve psychoeducation for families and healthcare providers to reduce stigma and promote social support, helping women seek help when needed.

Considering the high prevalence of depression (21.7%) and anxiety (22.2%) during pregnancy [13,14] and the evidence of adverse effects on mothers and children, it is essential to develop public health policies at all healthcare levels that promote early detection and effective management of these conditions. The prevalence of depression and anxiety during pregnancy is higher than in non-pregnant women of reproductive age; for example, a health survey in Nepal reported a depression prevalence of 5.4% (95% CI: 4.8% to 6.2%) and anxiety symptoms at 7.5% (95% CI: 6.7% to 8.4%). Additionally, 9.1% (95% CI: 8.2% to 10.1%) of women experienced either condition, while 3.8% (95% CI: 3.3% to 4.4%) experienced both. Women with lower education levels, more children, unemployed partners, and those living in rural areas showed higher rates of depression and anxiety symptoms [34]. This is similar to findings reported in a study using data from the National Health and Nutrition Examination Surveys 2007–2014 in the United States, which found that among 3705 non-pregnant women of childbearing age, the overall prevalences of major and minor depression were 4.8% and 4.3%, respectively [35].

In many developing countries, perinatal mental health remains an underrecognized area with limited visibility, low detection rates, and scarce resources. Estimating the prevalence of depression and anxiety during pregnancy is a critical step for raising awareness, guiding public policies, allocating resources, and improving maternal and child health outcomes [12,13,14].

There is limited data on prenatal depression and anxiety among the Mexican population; therefore, more research is necessary to examine the long-term effects of these emotional disorders during pregnancy and their impact on offspring health and neurodevelopment in childhood and adolescence.

It is also crucial to include qualitative methods to understand the psychosocial experiences of pregnant women better and to assess the role of protective factors such as social support, resilience, and partner involvement in reducing mental health symptoms during pregnancy.

## 5. Conclusions

Our results show that one in five pregnant women presented clinical symptoms of depression or anxiety, and one in eight experiences both conditions, emphasizing the importance of routine mental health screening and assessment during pregnancy. Factors linked to depression include anxiety, and having an education level of secondary or high school, while depression and having children are associated with anxiety.

## Figures and Tables

**Table 1:** Characteristics of participants included in the study.

Characteristic	Participants *n* = 288
Age (years)	30.2 ± 5.2
Pre-pregnancy body mass index	27.6 ± 5.4
Gestational age at enrollment (weeks)	13.0 ± 0.6
Edinburgh Postnatal Depression Scale	7.3 ± 6.1
State-Trait Anxiety Inventory	34.4 ± 10.2
Educational level	
Secondary school	61 (21.1%)
High school	122 (42.4%)
Bachelor’s degree	105 (36.5%)
Marital status	
Married	114 (39.6%)
Cohabiting	112 (38.9%)
Single	62 (21.5%)
Pre-pregnancy body mass index	
Normal (18.5–24.99 kg/m^2^)	107 (37.2%)
Overweight (25–29.99 kg/m^2^)	106 (36.8%)
Obesity (≥30 kg/m^2^)	75 (26.0%)

**Table 2:** Logistic regression analysis with adjusted odds ratios for variables associated with depression in the bivariate analysis.

Variable	Beta	*p*-Value	aOR (95% CI)
Married	−0.624	0.21	0.53 (0.20–1.41)
Single	0.624	0.21	1.86 (0.71–4.91)
Secondary and high school	0.957	0.03	2.60 (1.10–6.14)
Bachelor’s degree	−0.957	0.03	0.38 (0.16–0.90)
Anxiety	2.394	0.0001	10.8 (5.4–21.6)
Unplanned pregnancy	0.493	0.207	1.63 (0.76–3.52)
Having children	0.059	0.89	1.06 (0.45–2.45)
Nausea and vomiting > 3 times/week (1T)	0.444	0.25	1.55 (0.72–3.35)

aOR = Adjusted odd ratio; 95% CI = 95% confidence interval; 1T = First trimester.

**Table 3:** Logistic regression analysis with adjusted odds ratios for variables associated with anxiety in the bivariate analysis.

Variable	Beta	*p*-Value	aOR (95% CI)
Physical activity > 150 min/week	410	0.24	1.51 (0.75–2.77)
Primigravida	−0.041	0.93	0.96 (0.35–2.62)
Multigravida	0.041	0.93	1.04 (0.75–3.01)
Depression	2.351	0.0001	11.2 (5.6–22.1)
Having children	0.956	0.03	3.1 (1.60–6.10)
History of preterm birth	0.434	0.35	1.54 (0.62–3.84)
Nausea and vomiting > 3 times/week (1T)	0.333	0.34	1.39 (0.70–2.77)

aOR = Adjusted odd ratio; 95% CI = 95% confidence interval; 1T = First trimester.

## Data Availability

The datasets generated and analyzed during the present study are not publicly available but are available from the corresponding author on reasonable request.

## Abbreviations

The following abbreviations are used in this manuscript:

OBESO	Biochemical and Epigenetic Origins of Overweight and Obesity
INPerIER	National Institute of Perinatology Isidro Espinosa de los Reyes
EPDS	Edinburgh Postnatal Depression Scale
STAI	State-Trait Anxiety Inventory (STAI)
